# Improving Mechanical Properties of Carboxyl-Terminated Polybutadiene (CTPB) Binder System Using a Cure Accelerator

**DOI:** 10.3390/molecules31020284

**Published:** 2026-01-13

**Authors:** Xiao Qu, Peixuan Hu, Xinyi Ma, Yunfei Liu, Hongtao Yang, Wei Zhang, Yu Chen

**Affiliations:** 1 School of Materials Science and Engineering, Beijing Institute of Technology, Beijing 100081, China; qux2000@163.com (X.Q.); hupx97@163.com (P.H.); xinyima@sohu.com (X.M.); 2 Yangtze Delta Region Academy, Beijing Institute of Technology, Jiaxing 314019, China; 3 Xi’an Modern Chemistry Research Institute, Xi’an 710065, China; lyf3282@163.com (Y.L.); yht540@163.com (H.Y.)

**Keywords:** carboxyl-terminated polybutadiene, binder system, mechanical properties, cure accelerator

## Abstract

To address the issues of slow curing rate, post-curing reactions, and suboptimal mechanical properties in the carboxyl-terminated polybutadiene (CTPB)/epoxy resin (EP) binder system used for solid propellants, this study optimized the curing system by introducing 593 aliphatic amine compounds containing primary and secondary amine groups as a cure accelerator. It is found that the incorporation of the cure accelerator improved the fracture strength and elongation at break of the CTPB/EP binder system. With the addition of 0.3 wt.% cure accelerator, the tensile fracture strength increased to 0.37 MPa, while the elongation at break reached 655%. Moreover, augmenting the quantity of cure accelerator can substantially elevate the crosslink density and gel fraction of the binder system. When the addition reaches 0.3 wt.%, the crosslink density is 4.3 × 10^−4^ mol/cm^3^. Further studies showed that 593 cure accelerator reduced the activation energy of the curing reaction of the CTPB/EP binder system, with higher levels of cure accelerator resulting in lower activation energy. This study established a preparation methodology for a CTPB/EP binder system with high elongation and tensile strength. These findings provide a solid scientific foundation for the application of CTPB-based binder systems in solid propellants.

## 1. Introduction

Solid propellants are energetic composite materials composed of polymer binder, solid powder oxidizer, powdered metal fuel, and other additional components. This class of materials enables sustained and stable combustion, generating copious amounts of high-temperature and high-pressure gas, which in turn provides continuous thrust for propulsion systems [1]. The performance of solid propellant directly affects the operational effectiveness of rockets and missiles [2].

Carboxyl-terminated polybutadiene (CTPB) is predominantly synthesized via anionic or radical polymerization of 1,3-butadiene. It has been extensively applied as a polymer binder in solid rocket propellants due to its excellent adhesion, appropriate viscosity, mild curing reaction, and low heat release during curing [3,4]. Its curing reaction takes place on the terminal carboxyl group that can react with the curing agents containing epoxy aziridinium, alcohols, or isocyanate groups [5,6,7]. The reaction forms a curing network structure that can improve the casting molding process and mechanical properties of the cured binder and composite system [8,9]. Therefore, it is also a potential method to increase the solid content of the propellants and enhance the safety of preparation processes.

CTPB tends to absorb moisture, and the oxidizer is prone to produce CO_2_ and other small molecule by-products, which affects the density and uniformity of the cured propellant [10,11]. The curing system of CTPB and epoxy resin (EP) curing agents can form an ester bond network. The cross-linked products exhibit excellent mechanical properties and strength. As a result, epoxy resin curing agents are extensively utilized in CTPB binder system [12,13,14]. However, during the curing process of the EP and CTPB adhesive, the curing rate is relatively slow, resulting in a prolonged curing time. Additionally, the elongation at break of the solid propellant after curing is relatively low [15,16].

The carboxyl–epoxy reaction, although theoretically capable of proceeding without a cure accelerator, generally demands high temperatures to achieve adequate completion [17]. During the manufacturing process of solid propellants, the typical curing temperature range is 50–70 °C [18,19]. Surpassing this temperature threshold may induce thermal stress within the material. The energetic components in the propellant, including RDX (Royal Demolition Explosive) and AP (Ammonium Perchlorate), could encounter substantial safety hazards [20]. Utilizing end-amino terminated polybutadiene, which possesses higher reactivity, as the binder for epoxy resin, although eliminating the need for a cure accelerator, predominantly results in the formation of rigid C–N bonds within the resultant network [21,22]. This can enhance the brittleness of the material. Concurrently, an excessively rapid curing reaction rate can readily cause the viscosity of the mixed paste to escalate too swiftly. The solid fillers then encounter difficulties in achieving uniform dispersion, leading to localized aggregation of fillers and thereby engendering safety hazards [23,24].

To solve this problem, a substance capable of promoting the ring opening of the epoxy bond in EP can be added to facilitate the esterification between EP and the carboxyl group at the end of the CTPB moiety after ring opening. Aliphatic amine compounds, which contain amino groups, are capable of promoting the ring opening of EP under medium and low-temperature conditions. This characteristic makes them highly suitable for such applications and renders them ideal catalysts for the curing reaction between EP and CTPB [25,26,27].

**Figure 1:**
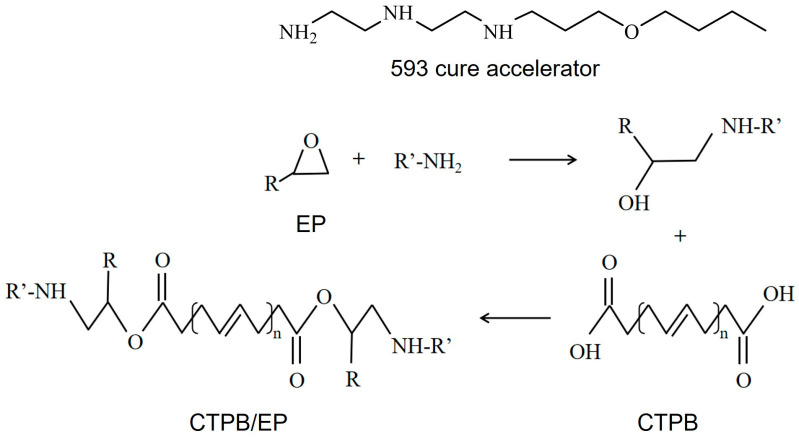
Cross-linking mechanism of carboxyl-terminated polybutadiene (CTPB)/epoxy resin (EP) binder system and molecular structure of each component.

For the CTPB/EP binder system in this article, EP is the curing agent, and dioctyl sebacate (DOS) is the plasticizer. To address the issues of low tensile strength and prolonged curing time in the CTPB/EP binder system, the 593 cure accelerator, which contains amino groups in its molecular structure, was employed in this study. The 593 cure accelerator is an adduct of diethylenetriamine and butyl glycidyl ether, offering advantages such as transparency, low toxicity, and low volatility [28]. The primary and secondary amine groups within this substance can facilitate the ring-opening reaction of the epoxy bonds in EP. Subsequently, the hydroxyl groups produced from the ring-opening reaction can engage in esterification reactions with carboxyl groups, thereby enhancing the curing efficiency and mechanical properties of the system. The cross-linking mechanism of the system and the molecular structure of each component are shown in Figure 1. The optimization of the formulation composition led to enhanced mechanical properties in the CTPB/EP binder system [29,30].

## 2. Results and Discussion

### 2.1. Infrared Spectroscopy (IR)

**Figure 2:**
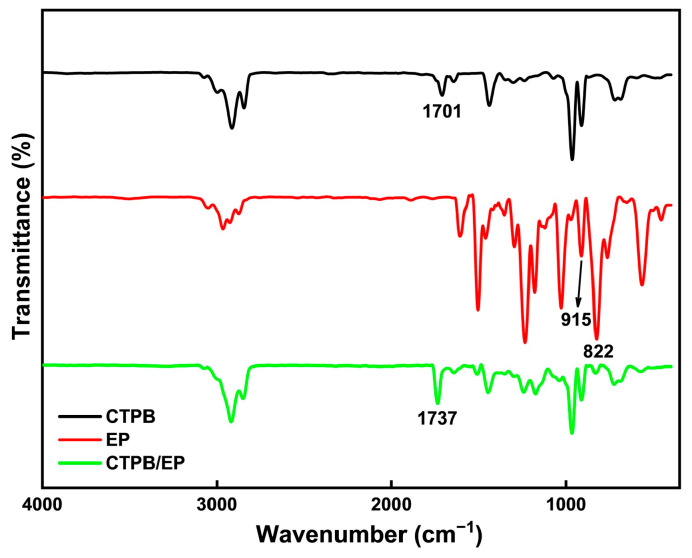
Infrared spectroscopy analysis of CTPB, EP, and CTPB/EP.

Figure 2 shows the Fourier Transform infrared spectroscopy (FTIR) spectra of CTPB, EP, and the cured CTPB/EP system with 4% EP content using the 593 cure accelerator. The spectral comparison clearly confirms the chemical reaction between CTPB and EP during curing. The pure EP spectrum displays characteristic epoxy group peaks at 915 cm^−1^ and 822 cm^−1^, corresponding to epoxy ring stretching vibrations. These peaks show substantial weakening in the cured product, indicating consumption of epoxy groups through ring-opening reactions. Similarly, the C=O stretching vibration of carboxyl groups in CTPB at 1701 cm^−1^ decreases significantly after curing, confirming the participation of –COOH groups in the reaction. A new peak appears at 1737 cm^−1^ in the cured product, characteristic of ester carbonyl groups. This provides direct evidence for ester bond formation between CTPB carboxyl groups and EP epoxy groups.

### 2.2. Mechanical Properties

**Figure 3:**
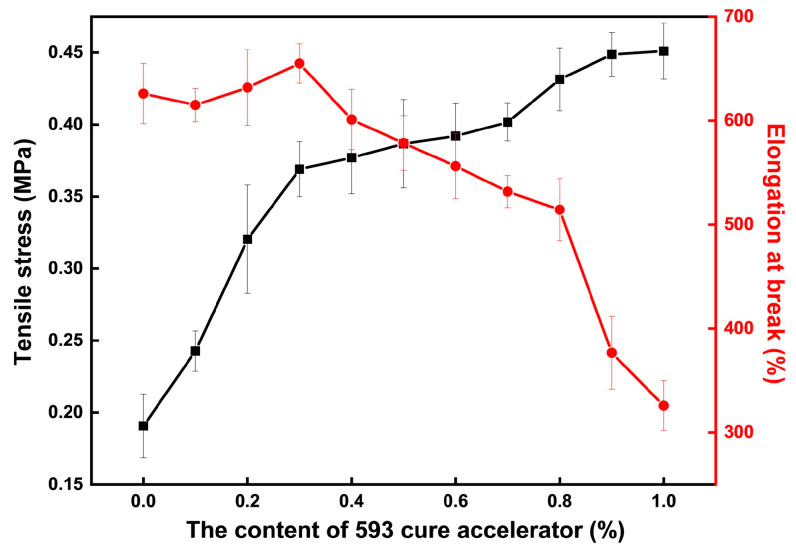
Changes in tensile strength and elongation at break of the CTPB/EP binder system with the increase in 593 cure accelerator content.

The mechanical properties test results of the CTPB/EP binder system with different amounts of cure accelerator are shown in Figure 3. The adhesive system without the addition of 593 cure accelerator has an elongation at break of 626% and a tensile strength of only 0.19 MPa. As the amount of 593 cure accelerator added increases, the tensile breaking strength of the CTPB/EP binder system gradually increases, while the elongation at break first slightly increases and then rapidly decreases. When an excessive amount of 593 cure accelerator is added, the toughness of the adhesive deteriorates.

When the amount of 593 cure accelerator added is 0.3 wt.%, the tensile breaking strength of the adhesive is 95% higher than that of the original adhesive, reaching 0.37 MPa, while achieving the highest elongation at break of 655%. Moreover, regarding the surface of the samples, the surface of the sample cured with the addition of 0.3 wt.% 593 cure accelerator is smoother than that of the adhesive sample without the addition of 593 cure accelerator.

Based on the above experimental observations, it can be inferred that the amine groups in the 593 cure accelerator can effectively catalyze the ring-opening reaction of the epoxy groups in EP and significantly enhance the reaction efficiency between the epoxy rings and the terminal carboxyl groups of CTPB, thereby promoting the formation of more ester bonds and ultimately creating a polymer network with a higher degree of cross-linking. The increase in crosslink density restricts the mobility of the polymer chains, enhances the material’s resistance to deformation, and thus improves the mechanical strength of the CTPB/EP binder system.

### 2.3. Crosslink Density

**Figure 4:**
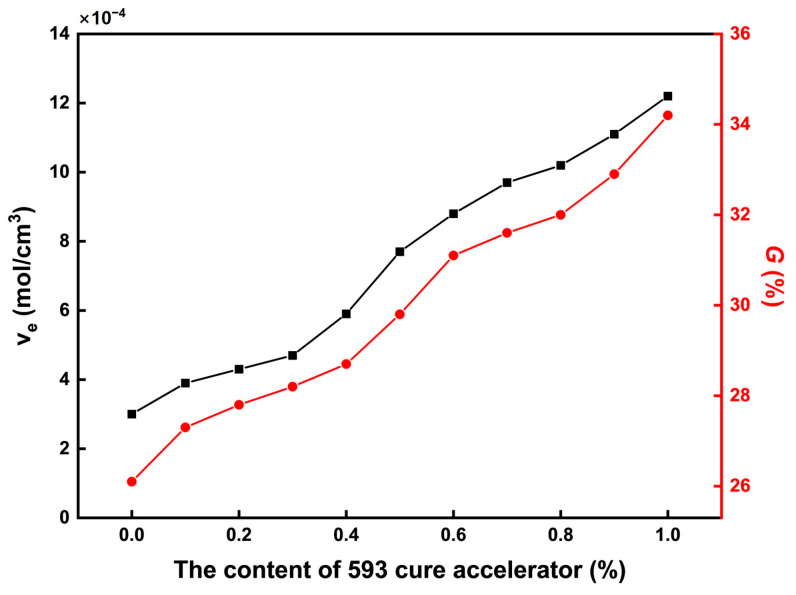
Variations in crosslink density and gel fraction of the CTPB/EP binder system with the increase in 593 cure accelerator content. $v_{e}$ is the crosslink density of the polymer (mol/cm^3^); G is related to the crosslinking network properties of the binder.

The cross-linking characteristics of the CTPB/EP binder systems were evaluated through crosslink density and gel fraction measurements, as summarized in Figure 4. Both parameters exhibit a clear increasing trend with higher contents of the 593 cure accelerator, indicating enhanced network formation. This trend can be attributed to the catalytic effect of the amine groups in the 593 cure accelerator. These amine groups can promote the ring-opening reaction of the epoxy groups in EP, thereby increasing the number of active sites in the system. The hydroxyl groups generated from the ring opening of EP can then form more ester bonds with the terminal carboxyl groups of CTPB, ultimately achieving an increase in crosslink density. The corresponding rise in gel fraction further confirms the formation of a more complete and robust three-dimensional network. This structural evolution corresponds to the observed increase in tensile breaking strength in the mechanical property tests of Section 2.2: Mechanical Properties.

### 2.4. Surface Morphology

**Figure 5:**
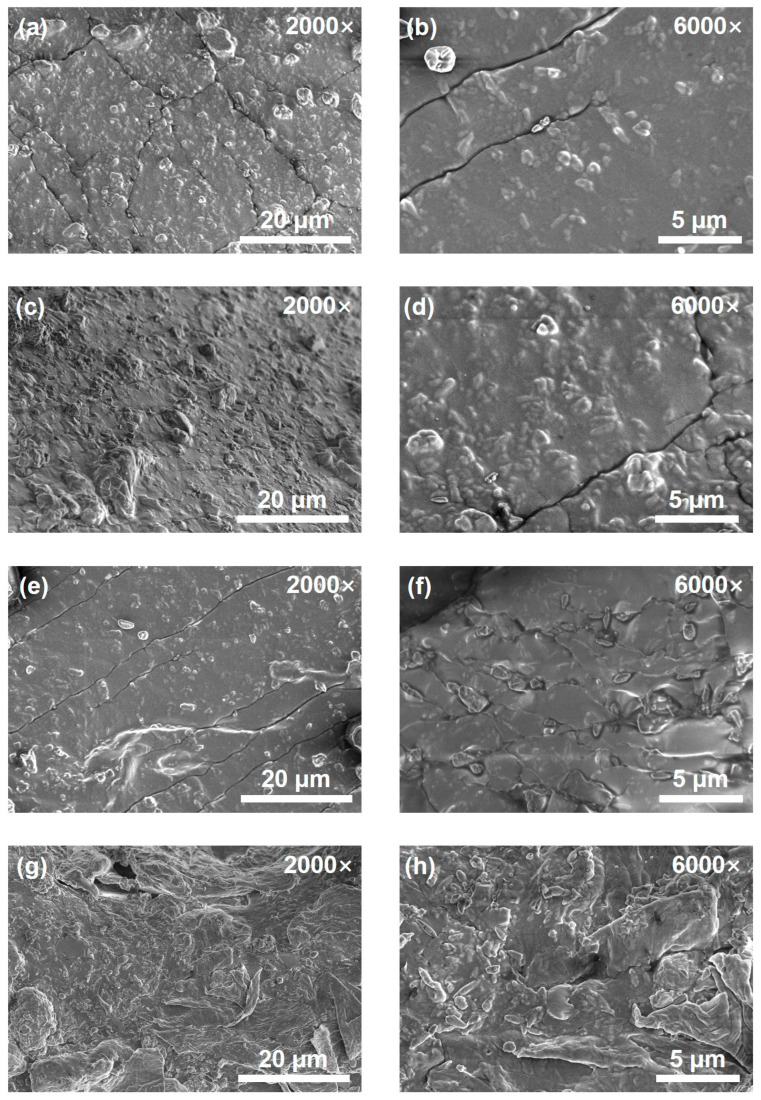
Scanning electron microscope (SEM) images of the CTPB/EP binder systems containing 0.3 wt.% (**a**,**b**), 0.5 wt.% (**c**,**d**), 1 wt.% (**e**,**f**) 593 cure accelerator and the control system sample (no 593 cure accelerator) (**g**,**h**). The left images are at a magnification of 2000×, and the right ones are at a magnification of 6000×.

Figure 5 shows the SEM images of the cured CTPB/EP binder systems with different 593 cure accelerator contents at the magnifications of 2000× and 6000×. As can be seen, the surface of the control binder system sample (no 593 cure accelerator) is extremely uneven (Figure 5g,h), and the solid filler is concentrated in the lower left corner of the sample, showing obvious signs of incomplete curing. Figure 5a–f illustrate the surface morphologies of the CTPB/EP binder systems containing 0.3 wt.%, 0.5 wt.%, and 1 wt.% of 593 cure accelerator, respectively. When the amount of cure accelerator added is low, obvious cracks appear on the surface of the sample. These cracks can absorb a certain amount of energy when the sample is stretched, thereby increasing both the tensile strength and the elongation at break of the system. As the 593 cure accelerator content increased, the binder surface gradually became smoother. The cracks decrease in number and tend to align in the same direction. The compact and uniform surface morphology can contribute to the higher axial tensile strength of the binder with higher cure accelerator contents. However, it also diminishes the toughness of the binder to certain extents, leading to lower elongations at break as observed macroscopically.

### 2.5. Effects of 593 Cure Accelerator on Curing Kinetics of CTPB Binder

**Figure 6:**
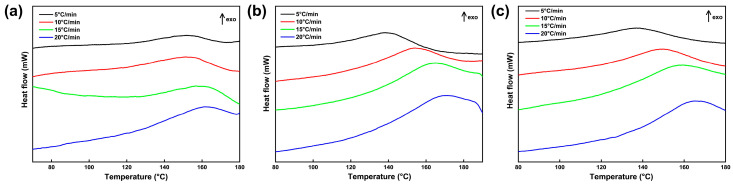
Differential scanning calorimetry (DSC) curves of CTPB/EP binder systems containing 0 wt.% (**a**), 0.5 wt.% (**b**), and 1 wt.% (**c**) 593 cure accelerator.

Studying the curing kinetics of the CTPB/EP binder system containing 593 cure accelerator is essential for understanding the affecting mechanism of the accelerator on the curing performance of CTPB binder and enabling the selection of optimal reaction conditions and controllable reaction process. It is of significant importance for tuning the binder network structure, predicting the pot life of the slurry, and preparing propellants that meet the requirements in mechanical and process properties. For the curing reaction kinetics analysis, three CTPB/EP binder systems containing 0 wt.%, 0.5 wt.%, and 1 wt.% 593 cure accelerators were prepared and subjected to mechanical testing and cross-linking network structure evaluation. Figure 6 displays the non-isothermal differential scanning calorimetry (DSC) curves of the three CTPB/EP binder systems at different heating rates. The characteristic peak temperatures of the DSC curves are listed in Table 1.

As can be seen from Figure 6 and Table 1, the corresponding exothermic peaks of all three binder systems shift to the high temperature region, and the peak shapes gradually become sharper with the increase in the heating rate. The cross-system comparison reveals that the temperature difference of the peak temperature ($T_{p}$) between the different heating rates gradually increases with the increase in 593 cure accelerator content. This can be explained by the strong curing promotion capability of 593 cure accelerator. Higher cure accelerator contents shorten the curing time. At lower cure accelerator contents, the phase transition from liquid to solid is longer, and thus the heat release is higher at a constant heat release rate during the exothermic curing reaction. Consequently, the peak temperature difference between low and high heating rates is smaller. The phase transition process at higher cure accelerator contents is fast and the curing time is shorter, which produces less heat dissipation during the process. Because the thermal conductivity of a solid is lower than that of a liquid, the shorter time and higher insulation rate result in larger temperature differences between low and high heating rates, while the exothermic heat release remains constant. This phenomenon clearly demonstrates the strong curing promotion effect of 593 cure accelerator on the CTPB/EP binder system.

**Figure 7:**
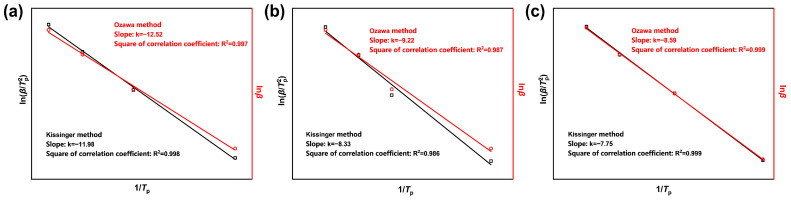
Curing kinetics fitting curves of CTPB/EP binder systems containing 0 wt.% (**a**), 0.5 wt.% (**b**), and 1 wt.% (**c**) 593 cure accelerator. β is the heating rate (°C/min); $T_{p}$ is the peak temperature (°C).

The non-isothermal curing kinetics parameters of the CTPB/EP binder system were determined by the linear fitting of $ln(\beta /{T_{p}}^{2})\;$ against $1/T_{p}$ using the Kissinger method and the linear fitting of lnβ against $1/T_{p}$ using the Ozawa method [31]. The $T_{p}$ corresponding to the DSC curves of the three formulas at different heating rates were obtained from Table 1. The fitting results are shown in Figure 7, and the kinetic parameters obtained by the two fitting methods are presented in Table 2. The activation energies obtained by the two methods were then calculated and averaged.

The apparent activation energy of the curing reaction is higher for the binder system in the absence of 593 cure accelerator. The reaction rate is low at low temperatures and only significantly increases above the critical temperature. After the addition of the 593 cure accelerator, the average activation energy shows a decreasing trend with the increase in the cure accelerator content. This suggests that the 593 cure accelerator can effectively lower the energy barrier of the curing reaction of the CTPB/EP binder, making the reaction occur more easily and thereby increasing the reaction rate. This explanation, from the perspective of curing kinetics, elucidates the differences in the curing rate during the macroscopic curing process. The activation energy parameters obtained can provide strong support for the optimization of curing conditions and the regulation of the network structure.

## 3. Materials and Methods

### 3.1. Materials

Carboxy-terminated polybutadiene (CTPB; the carboxyl value is 0.588 mmol/g) was purchased from Liming Research Institute of Chemical Industry (Luoyang, China). Bisphenol A-type epoxy resin (E-51; epoxy equivalent: 184~195 g/mol) was purchased from Shandong Yousuo Chemical Science and Technology Co., Ltd. (Heze, China). The 593 cure accelerator (hydroxyl value of 500~700 mg·KOH/g) was purchased from Guangzhou Yuanshengtai Chemical Co., Ltd. (Guangzhou, China). Dioctyl sebacate (DOS) and toluene were all analytical-grade. DOS was purchased from Zhengzhou Baotai Nanomaterials Co., Ltd. (Zhengzhou, China), and toluene was purchased from Shanghai Zhixin Chemical Co., Ltd. (Shanghai, China).

### 3.2. Preparation of CTPB/EP Binder Systems

The formulas of different CTPB/EP binder systems are shown in Table 3. For a typical preparation process, E-51 was preheated in an oven at 70 °C for 20 min for better fluidity and mixed with a certain amount of CTPB. The mixture was put on a thermostatic stirrer of 70 °C and stirred at 300 rpm for 20 min until the system became milky white. After thorough stirring, the mixture was slowly poured into a polytetrafluoroethylene mold, transferred into a vacuum oven of 70 °C to remove air bubbles, and cured at 75 °C for 5–7 days.

### 3.3. Measurements and Characterizations

#### 3.3.1. Fourier Transform Infrared Spectroscopy (FTIR) Testing

FTIR analysis of the binder samples was conducted using a Nicolet 8700 spectrometer (Thermo Fisher Scientific, Waltham, MA, USA) in ATR mode. Spectra were collected in the range of 4000 to 400 cm^−1^ at a resolution of 4 cm^−1^ with 64 scans.

#### 3.3.2. Mechanical Testing

The obtained binder films were cut into dumbbell-shaped specimens (GBT528-2009 [32], Type 3) for the mechanical tests on an Instron 6022 general-purpose material testing machine (Instron, Norwood, MA, USA). The tensile tests were conducted at 25 °C at a tensile strain rate of 100 mm/min. Each test was repeated three times, and the mean value and standard deviation were calculated and recorded.

#### 3.3.3. Crosslink Density Measurement

The cross-link densities of the cured binder films were determined by the density bottle method. A sample of approximately 0.25–0.35 g was cut from the binder film, accurately weighted as *m*_0_, and then immersed in 50 mL of toluene at 25 °C for 24 h. Fresh toluene was then added to continue the dissolution process for additional 48 h. The dissolved film was swiftly dried with filter paper and weighed, and the mass was recorded as *m*_s_. The film sample was then dried thoroughly to remove the toluene and weighed again, and the mass was recorded as *m*_d_.

According to the Flory–Huggins polymer solution theory and the rubber entropy elasticity theory, for the dissolution of a polymer in a small-molecule solution, the maximum degree of dissolution is reached when its tendency to dissolve and elastic contraction reach the equilibrium state. In this state, the crosslink density of the polymer can be calculated with the Flory–Rehner equation as follows [33,34].

(1) $$v_{e}=-\frac{\left[\ln\left(1-V\right)+V+\chi \cdot V\right]}{V_{s}\cdot \left(V^{1/3}-2V/f\right)}=\frac{\rho_{e}}{\bar{M_{c}}}$$

where $v_{e}$ is the crosslink density of the polymer, mol/cm^3^; $\bar{M_{c}}$ is the average molecular weight between crosslinking points, g/mol; V is the volume fraction of the polymer swollen in the solvent; $V_{s}$ is the molar volume of the solvent; χ is the interaction parameter of the polymer with the solvent; f is the functional degree of the polymer crosslinking network; and $\rho_{e}$ is the density of the polymer.

V can be calculated with Equation (2):

(2) $$V=\frac{m_{d}/\rho_{e}}{m_{d}/\rho_{e}+({m_{s}-m}_{d})/\rho_{s}}$$

where $\rho_{s}$ is the density of the solvent.

χ can be obtained with the Bristow–Watson semi-empirical equation:

(3) $$\chi =0.34+\frac{V_{s}}{RT}\cdot {\left(\delta_{s}-\delta_{e}\right)}^{2}$$

where R is the ideal gas constant; T is the absolute temperature; and $\delta_{s}$ and $\delta_{e}$ are the solubility parameters of the solvent and polymer respectively. The latter can be estimated by the group contribution method [35,36].

The gel fraction G refers to the proportion of the macromolecular gel produced by the binder system in the total system after curing [37]. Under certain conditions, G is related to the crosslinking network properties of the binder. It can be calculated as

(4) $$G=\frac{m_{d}}{m_{0}}\cdot 100\%$$

#### 3.3.4. Micro-Morphological Examination

The surface morphologies of the obtained binder films were imaged using a MIR-AXM cold field emission scanning electron microscope (TESCAN, Brno, Czech Republic). The samples were sprayed with a layer of gold before testing, and the accelerating voltage was set to 3.5 kV.

#### 3.3.5. Curing Kinetics Testing

Three uncured CTPB binder formulas, each containing 10 mg CTPB and a certain amount of 593 cure accelerator, were accurately weighed, placed in a covered aluminum crucible in a N_2_ atmosphere, and allowed to cure at heating rates of 5 °C/min, 10 °C/min, 15 °C/min, or 20 °C/min. The DSC curves during the curing process were recorded using a 200 F3 differential scanning calorimeter (NETZSCH, Selb, Germany) to investigate the influences of the cure accelerator on the kinetics of CTPB curing reaction. The heating program consisted of three sections: the temperature ramping from room temperature to 200 °C at the preset heating rate to eliminate thermal history, the temperature being held at 200 °C for 30 min to ensure a complete reaction, and temperature decreasing from 200 °C to room temperature at the preset heating rate. The initial temperature ($T_{i}$), peak curing temperature ($T_{p}$), and final temperature ($T_{f}$) of the reaction at each heating rate were recorded. The apparent activation energy ($E_{a}$) is a measure of the minimum energy required for the resin to initiate the curing reaction, and its magnitude is one of the crucial factors in determining the curing reaction rate. The Kissinger Equation (5) and the Ozawa Equation (6) are the commonly used calculation methods of the activation energy for the curing reaction of thermosetting resins [38,39]. In this study, the curing reaction activation energy of each system was calculated by both the Kissinger method and Ozawa method, and the initial temperature, the peak curing temperature, and the post-curing treatment temperature of each system were determined by the T-β extrapolation method [40,41].

(5) $$\mathrm{Kissinger}\;\mathrm{equation}:\;E_{a}=-R\frac{dln\left(\beta /T_{p}^{2}\right)}{d\left(1/T_{p}\right)}$$

(6) $$\mathrm{Ozawa}\;\mathrm{equation}:\;E_{a}=-0.95R\frac{dln\left(\beta \right)}{d\left(1/T_{p}\right)}$$

where β is the heating rate (°C/min); R is the molar gas constant with a value of 8.314 J/(mol·K).

## 4. Conclusions

To tackle the challenges of slow curing rate and suboptimal mechanical properties in the CTPB/EP binder system used for solid propellants, this study explored the impact of varying amounts of 593 cure accelerator—an aliphatic amine compound—on adhesive properties. The experimental results demonstrated that when the amount of 593 cure accelerator added was 0.3 wt.%, the tensile breaking strength of the adhesive increased by 95% compared to the original adhesive, reaching 0.37 MPa, while achieving the highest elongation at break of 655%. The crosslink density and gel fraction of the cured adhesive also gradually increased. The activation energy calculations for the curing reaction of the CTPB/EP binder system revealed that the 593 cure accelerator could significantly reduce the energy required for the curing reaction. In summary, this study clarified the preparation conditions for a CTPB/EP binder system with both high elongation at break and high tensile breaking strength. By adjusting the formulation, controllable regulation of the curing time and mechanical properties of the system was achieved.

This study achieved accelerated curing of the binder without increasing the curing temperature, thereby reducing the production cycle of solid propellant and significantly enhancing industrial production efficiency. Together, the relatively low curing temperature and optimized curing rate avoided filler aggregation, reduced thermal hazards, and enhanced process safety. Consequently, this work provides a solid research foundation for the application of this system in solid propellants.

## Figures and Tables

**Table 1:** Thermal curing reaction characteristic temperatures of the CTPB/EP binder systems at different heating rates.

Addition of 593 (wt.%)	β (°C/min)	*T_i_* (°C)	*T_p_* (°C)	*T_f_* (°C)
0	5	81.4	150.5	171.6
	10	82.3	154.2	174.5
	15	84.7	159.8	176.5
	20	85.4	163.5	181.3
0.5	5	83.1	138.4	167.9
	10	85.4	154.2	173.3
	15	81.3	161.3	181.4
	20	82.7	166.5	184.4
1.0	5	83.2	136.7	167.8
	10	84.6	150.6	171.3
	15	88.7	159.8	178.4
	20	81.3	165.4	180.4

β is the heating rate (°C/min). $T_{i}$ is the initial temperature (°C); $T_{p}$ is the peak curing temperature (°C); $T_{f}$ is the final temperature (°C).

**Table 2:** Non-isothermal curing kinetic parameters of CTPB/EP binder systems.

Addition of 593 (wt.%)	E_a_(1)/kJ∙mol^−1^	E_a_(2)/kJ∙mol^−1^	ΔE_a_/kJ∙mol^−1^	A/s^−1^
0	99.60	98.95	99.28	1.01 × 10^9^
0.5	69.26	72.87	71.07	1.47 × 10^5^
1.0	64.43	67.89	66.16	3.73 × 10^4^

E_a_(1) is the apparent activation energy used the Kissinger calculation method, and E_a_(2) is the apparent activation energy used the Ozawa calculation method. ΔE_a_ is the average value of E_a_(1) and E_a_(2). A is the pre-exponential factor.

**Table 3:** Formulas of CTPB curing systems.

Sample Number	CTPB (wt.%)	E-51 (wt.%)	DOS (wt.%)	593 (wt.%)
1	56	9.0	35	0.0
2	56	8.9	35	0.1
3	56	8.8	35	0.2
4	56	8.7	35	0.3
5	56	8.6	35	0.4
6	56	8.5	35	0.5
7	56	8.4	35	0.6
8	56	8.3	35	0.7
9	56	8.2	35	0.8
10	56	8.1	35	0.9
11	56	8.0	35	1.0

## Data Availability

The original contributions presented in this study are included in the article. Further inquiries can be directed to the corresponding author. Yu Chen can be contacted to request the data at cylsy@163.com.
